# Gut Microbiome-Mediated Alteration of Immunity, Inflammation, and Metabolism Involved in the Regulation of Non-alcoholic Fatty Liver Disease

**DOI:** 10.3389/fmicb.2021.761836

**Published:** 2021-11-02

**Authors:** Li-Hong He, Dun-Han Yao, Ling-Yun Wang, Lei Zhang, Xue-Li Bai

**Affiliations:** ^1^Department of Hepatobiliary and Pancreatic Surgery, the First Affiliated Hospital, School of Medicine, Zhejiang University, Hangzhou, China; ^2^The First Clinical Medical College, Lanzhou University, Department of General Surgery, The First Hospital of Lanzhou University, Lanzhou, China

**Keywords:** gut microbiota, non-alcoholic fatty liver disease, immune, metabolism, gut–liver axis

## Abstract

Non-alcoholic fatty liver disease (NAFLD) is one of the leading causes of end-stage liver disease, leading to a rapidly growing global public health burden. The term “gut microbiome (GM)” refers to the approximately 100 trillion microbial cells that inhabit the host’s gastrointestinal tract. There is increasing evidence that GM is involved in the pathogenesis of NAFLD and may be a potential target for intervention. To explore GM-based strategies for precise diagnosis and treatment of NAFLD, great efforts have been made to develop a comprehensive and in-depth understanding of the host–microbe interaction. This review evaluates this interaction critically, mainly considering the intricate regulation of the metabolism, immunity, and inflammatory status during the evolution of the disease pathogenesis, revealing roles for the GM in NAFLD by examining advances in potential mechanisms, diagnostics, and modulation strategies.

**Synopsis:** Considering the intricate metabolic and immune/inflammatory homeostasis regulation, we evaluate the latest understanding of the host–microbe interaction and reveal roles for the gastrointestinal microbiome in NAFLD. Strategies targeting the gastrointestinal microbiome for the diagnosis and treatment of NAFLD are proposed.

## Introduction

Non-alcoholic fatty liver disease (NAFLD) describes a collection of hepatic clinicopathological syndromes that range from simple hepatic steatosis, non-alcoholic steatohepatitis (NASH) to fat-related fibrosis and cirrhosis (Brunt et al., 2015). Characterized by excessive fat accumulation without a definite liver damaging factor, NAFLD is an acquired metabolic stress liver injury closely related to obesity, insulin resistance, and genetic susceptibility (Brunt et al., 2015; Wang and Malhi, 2018). Resulting from continuous damage to hepatocytes, the incidence of hepatocellular carcinoma (HCC) in patients with NAFLD is much higher than that in healthy people (Ipsen et al., 2018; Younossi et al., 2019). In some western countries, NAFLD has become the fastest-growing cause of HCC (Huang et al., 2021). With the prevalence of obesity and metabolic syndrome, NAFLD has become a major chronic liver disease worldwide, causing a global public health concern (Younossi et al., 2016; Huang et al., 2021). Considering its high morbidity, poor prognosis, and the lack of targeted and effective drugs, strategies to prevent and treat NAFLD are urgently needed to reduce the increasing burden of the disease.

The human gastrointestinal microbiome (GM) refers to the assemblage of microorganisms (e.g., bacteria, fungi, viruses, and protozoans) that inhabit the gastrointestinal tract (Sender et al., 2016). The results of metagenomic sequencing indicated that the GM comprises more than 1,000 kinds of microorganisms, affected by genetics, eating habits, and environmental factors (Zhernakova et al., 2016). A balanced GM plays a beneficial role in the physiological regulation of the host by balancing local and systemic immune responses, maintaining normal gut–liver circulation, and inhibiting pathogen colonization. Dysbacteriosis will lead to various diseases (e.g., metabolic diseases, immune diseases, respiratory diseases, and even tumors; Hand et al., 2016; Gong et al., 2018; Schirmer et al., 2018; Canfora et al., 2019; He et al., 2020). In particular, considerable research has demonstrated that the GM and its metabolites potentially affect the occurrence and prognosis of NAFLD by participating in the host’s immune and inflammatory responses, and nutrient intake and metabolism (Aron-Wisnewsky et al., 2020; Hu et al., 2020). Similarly, dysbacteriosis and the resulting increased gut inflammation and weakened immune surveillance play pivotal roles in leading to NASH, cirrhosis, and NAFLD-related HCC (Tripathi et al., 2018; Ezzaidi et al., 2019; Albillos et al., 2020).

In the present review, we dissect the role of the GM and their inflammatory mediators on immune regulation in NAFLD. Specifically, we focus on the characteristic changes of the GM in patients with NAFLD, including diversity and uniformity/homogeneity, and the developed non-invasive diagnostic strategies. In addition, the mechanism by which the GM regulates metabolic and immune homeostasis during the onset and progression of NAFLD and advances in modulating the GM to treat NAFLD are also highlighted.

## The Gut–Liver Axis and the Intestinal Barrier

**Figure 1:**
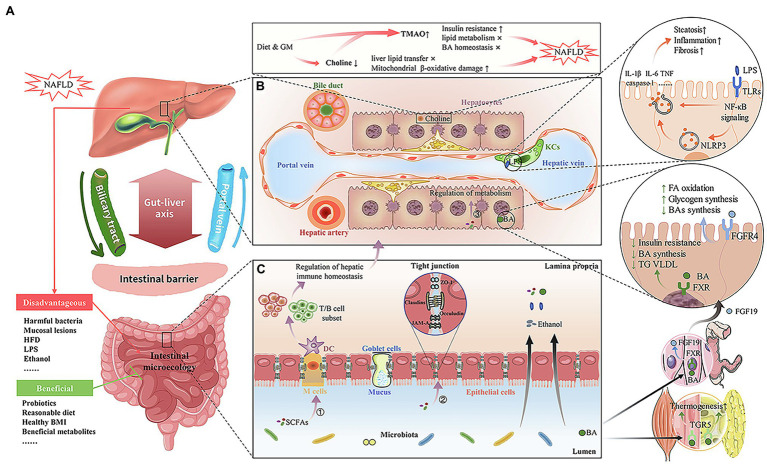
Interaction between the intestinal flora, its metabolites, and Non-alcoholic fatty liver disease (NAFLD). **(A)** Gut–liver axis. Based on the portal vein and biliary system, there is an interaction between the liver and the gut/gastrointestinal microbiome (GM). A healthy intestinal barrier and intestinal microecology are beneficial to maintain liver health. The disadvantageous factors include NAFLD, harmful bacteria, mucosal lesions, HFD, lipopolysaccharide (LPS), and ethanol. The beneficial factors include probiotics, reasonable diet, healthy BMI, and beneficial metabolites. **(B)** The liver microenvironment and GM-derived metabolites associated with NAFLD. Choline deficiency and TMAO increase caused by diet and GM disorders can lead to or aggravate NAFLD by enhancing insulin resistance and lipid metabolism disorder. The transfer of excessive LPS, secondary to intestinal barrier damage, to the liver will interact with TLRs on Kupffer cells and stellate cells, thus activating NF-κB and NLRP3 signaling pathways to produce hepatic pro-inflammatory and pro-fibrotic mediators, which aggravates NAFLD. The nuclear receptor, FXR, is activated by BAs in the liver and has several downstream effects, such as inhibition of TG, VLDL, and BA synthesis, and increased insulin sensitivity. BAs also activate TGR5 in muscle and adipose tissues, thereby increasing thermogenesis and energy expenditure. In the terminal ileum, and after BA uptake by the ileal apical sodium-dependent bile acid transporter, FXR also stimulates production of FGF19, which, upon binding to FGFR4 in liver cells, represses BA synthesis and promotes hepatic glycogen storage and FA oxidation. **(C)** The gut microenvironment and GM-derived metabolites associated with NAFLD. The intestinal barrier, including intestinal epithelial cells and tight junctions, plays an important role in intestinal tract permeability. When the intestinal barrier is destroyed, intestinal permeability will increase, and then the bacteria and their metabolites (e.g., endogenous alcohol) will transfer to the liver and eventually cause or aggravate NAFLD. SCFA is the most important metabolite of intestinal bacteria, which affects liver health in three ways, including immunity, nutrient intakeand metabolism, and tight junction function.

Many studies have demonstrated cross-talk between the GM and multiple organs of the host, which affects local and systemic metabolism and immune homeostasis (Hand et al., 2016; Gong et al., 2018; Schirmer et al., 2018; Canfora et al., 2019; He et al., 2020). The interaction among the gut, its contents, and the liver is called the “gut–liver axis,” resulting from the integrated signals generated by genes, diet, and environmental factors (Figure 1A; Tripathi et al., 2018). The portal vein and biliary system are the basis of this bidirectional interaction. On the one hand, the portal vein can transport intestinal origin immune cells, cytokines, and gut-derived products directly to the liver, such as secondary bile acids (BAs), short chain fatty acids (SCFAs), and lipopolysaccharide (LPS). On the other hand, the liver can secrete bile and many bioactive mediators into the intestine through the biliary system (Tripathi et al., 2018; Albillos et al., 2020). The interdependence between the liver and the gut explains why intestinal barrier damage can lead to some components of the microbiota and their metabolites flowing into the liver, leading to or aggravating a series of liver diseases.

The intestinal barrier in the gut–liver axis includes physical, immune, and biochemical components, plays an important role in the gut–liver axis (Peterson and Artis, 2014). The gut vascular and single layer epithelial cells, linked by tight junction proteins, together with the mucus layer and microorganisms, constitute a physical barrier. Molecules with antimicrobial properties, such as BAs and antimicrobial proteins, maintain and mediate biochemical barriers. Secreted immunoglobulin A (SIgA) and lymphoid follicles containing a variety of immune cells are the main parts of the immune barrier. In the normal physiological state, the intestinal barrier constitutes the first line of defense in human immunity, while the liver provides the second line of defense for pathogenic factors that escape from the intestinal mucosal immune defense; the immune tissues in the intestine and liver participate in the immune tolerance to food antigens and the clearance of pathogens (Martens et al., 2018).

Gastrointestinal microbiome dysbiosis can disrupt these barriers, increasing mucosal permeability. Dietary factors can not only alter the intestinal microbiome composition, but also play a vital role in the maintenance of the intestinal barrier. The pathological state of the intestinal barrier induced by a high fat diet (HFD) results in intestinal bacterial translocation and endotoxin entering the portal venous system (De Santis et al., 2015). As feedback, immune cells in the liver are activated by these pathogenic factors, releasing host inflammatory factors, and resulting in tissular damage to the intestinal mucosa, liver, and systemic organs (Szabo, 2015). The GM and its metabolites have a range of effects on the health and disease of liver, and the methods to promote optimum liver health are a major concern.

## Intestinal Microecology Disorder in Patients with Nafld

The characteristics of the GM in patients have both homogeneity and heterogeneity (Table 1). Wang et al. (2016) observed a lower diversity and a phylum-level change in the GM in patients with NAFLD. Compared with those in the healthy control group, the patients had 20% more Bacteroidetes and 24% less Firmicutes. Notably, the abundances of four families of Firmicutes were decreased significantly, including *Lachnospiraceae*, *Ruminococcaceae*, *Lactobacillaceae*, and *Peptostreptococcaceae*, which are SCFA-producing and 7α-dehydroxylating bacteria (Wang et al., 2016). In a prospective cross-sectional study, the abundances of *Ruminococcus*, *Paucalinbacterium prausnitzii*, and *Coprococcus* in patients with NAFLD were lower than those in healthy people; the difference was independent of body-mass index and insulin resistance (Da Silva et al., 2018). Interestingly, patients with NAFLD have higher fecal concentrations of propionic acid and isobutyric acid, and higher serum concentrations of 2-hydroxybutyric acid and L-lactic acid, than healthy controls (Da Silva et al., 2018). A cohort study in China showed that 60% of patients with NAFLD had a high abundance of *Klebsiella pneumoniae* (alcohol high-producing; Yuan et al., 2019). In the mouse model, a specific *K. pneumoniae*-rich microbiota isolated from patients with NAFLD and transplanted into healthy mice could aggravate liver inflammation and induce NAFLD (Yuan et al., 2019), which implied endogenous alcohol produced by these bacteria is an important pathogeny of NAFLD.

**Table 1:** Research on the changes of GM and metabolites in patients with NAFLD.

Subjects	Disease	Alteration of microbiota and metabolites	References
Mice	NAFLD	**Phylum:** Bacteroidetes↑ **Family:** Prevotellaceae↑ **Genus:** *Lactobacillus*↓	Henao-Mejia et al., 2012
		**Phylum:** Firmicutes↑ *Allobaculum*↓, *Barnesiella*↑, *Roseburia*↑ **Species:** *Bacteroides vulgatus*↓, *Lachnospiraceae bacterium*↑, *Barnesiella intestinihominis*↑	Le Roy et al., 2013
		**Species:** *Lactobacillus gasseri*↑, *Lactobacillus taiwanensis*↑	Zeng et al., 2013
		**Family:** Enterobacteriaceae↑ *Bifidobacteria*↓	Cano et al., 2013
Children	NASH	**Phylum:** Actinobacteria↓, Firmicutes↓, Proteobacteria↑, Bacteroidetes↑ Bifidobacteriaceae↓, Rikenellaceae↓, Lachnospiraceae↓, Ruminococcaceae↓, Prevotellaceae↑ *Bifidobacterium*↓, *Alistipes*↓, *Blautia*↓, *Escherichia coli*↑, *Prevotella*↑	Zhu et al., 2013
	NAFLD	**Family:** Gammaproteobacteria↑ *Prevotella*↑	Michail et al., 2015
		α-Diversity↓ 1-pentanol and 2-butanone↑ Actinobacteria↑, Bacterioidaceae↓ Rikenellaceae↓ *Ruminococcus*↑, *Bradyrhizobium*↑, *Oscillospira*↓; *Anaerococcus*↑, *Peptoniphilus*↑, *Dorea*↑, *Propionibacterium acnes*↑	Del Chierico et al., 2017
Adult	NAFLD	**Class:** Gammaproteobacteria↓, Erysipelotrichia↑	Spencer et al., 2011
		**Phylum:** Firmicutes↓, Actinobacteria↓, Bacteroidetes↑ *Blautia*↓, *Faecalibacterium*↓, *Bifidobacterium*↓, *Prevotella*↑	Zhu et al., 2013
		**Phylum:** *Firmicutes*↑; *Lactobacillaceae*↑, *Lachnospiraceae*↑, *Ruminococcaceae*↓, *Veillonellaceae*↑, *Kiloniellaceae*↑, *Pasteurellaceae*↑, *Porphyromonadaceae*↓	Raman et al., 2013
		**Family:** *Lactobacillaceae*↑, *Lachnospiraceae*↑, *Ruminococcaceae*↓ *Lactobacillus*↑, *Robinsoniella*↑, *Roseburia*↑, *Dorea*↑, *Oscillibacter*↓	Mouzaki et al., 2013
		**Genus:** *Alistipes*↓, *Prevotella*↓, *Escherichia coli*↑, *Odoribacter*↓, *Lactobacillus*↑, *Oscillibacter*↓, *Anaerobacter*↑, *Clostridium XI*↑, *Streptococcus*↑, *Flavonifractor*↓	Jiang et al., 2015
		**Phylum:** *Firmicutes*↓, *Bacteroidetes*↑	Wang et al., 2016
		**Phylum:** *Actinobacteria*↑, *Bacteroidetes*↓	Del Chierico et al., 2017
		**Phylum:** *Firmicutes*↓, *Proteobacteria*↑ *Escherichia coli*↑, *Bacteroides vulgatus*↑	Loomba et al., 2019
		**Phylum:** *Proteobacteria*↑, *Fusobacteria*↑ *Lachnospiraceae*↑, *Enterobacteriaceae*↑, *Erysipelotrichaceae*↑,*Streptococcaceae*↑ *Shigella*↑, *Prevotella*↓	Shen et al., 2017
		**Phylum:** *Firmicutes*↑; *Porphyromonas*↑, *Odoribacter*↓, *Succinivibrio*↑, *Proteus*↓	Li et al., 2018
		α/β Diversity↓; primary and secondary BAs↑ *Fusobacteria*↑ *Ruminococcaceae*↓ *Oscillospira*↓, *Ruminococcus*↓, *Coprococcus*↓;	Kim et al., 2019
		**Species:** *Prevotella copri*↑	Schwimmer et al., 2019
		**Phylum:** *Firmicutes*↓, *Bacteroidetes*↑ *Clostridia*↓	Tsai et al., 2020
	NASH	**Phylum:** *Proteobacteria*↑ *Enterobacteriaceae*↑ *Escherichia*↑	Zhu et al., 2013
		**Genus:** *Faecalibacterium*↓, *Anaerosporobacter*↓, *Parabacteroides*↑, *Allisonella*↑	Wong et al., 2013a
		**Species:** *Ruminococcus*↑, *Blautia*↑, *Dorea*↑, *Oscillospira*↓	Del Chierico et al., 2017
		**Family:** *Enterobacteriaceae*↑ *Akkermansia muciniphila*↓, *Bifidobacterium infantis*↑, *Lactobacillus reuteri*↑	Ozkul et al., 2017
		**Phylum:** *Bacteroidetes/Firmicutes* ratio↑ *Prevotella*↑	Sobhonslidsuk et al., 2018
		**Phylum:** *Bacteroidetes*↓ *Clostridium coccoides*↑	Mouzaki et al., 2013
		**Family:** *Bacteroidaceae*↑, *Prevotellaceae*↓ *Bacteroides*↑, *Prevotella*↓	Boursier et al., 2016

Most patients with NAFLD are obese but some patients belong to “lean NAFLD,” its pathogenesis remaining unclear. A recent study showed that the lean NAFLD group has a more Dorea and total BAs, but a fewer *Marvinbryantia* and *Christensellenaceae R7*, compared with lean healthy control, which provides an insight into microbial drivers of lean NAFLD pathogenesis (Younes and Bugianesi, 2019; Chen et al., 2020).

These findings indicated the potential role of specific microbiota and the characteristics of its metabolites in the pathogenesis of NAFLD. Based on these characteristics, the GM might be used as a non-invasive biomarker of NAFLD phenotype and provide prognostic value in the risk of progression to cirrhosis and HCC (Table 2).

**Table 2:** Clinical research on the diagnosis of NAFLD and related diseases by targeting GM.

Disease	Diagnostic tool and mechanism	References
NAFLD-liver fibrosis	Based on the specific differences in microbiota and BAs in both blood and feces that correlate with the presence of liver fibrosis	Lelouvier et al., 2016
NAFLD-liver fibrosis	Based on a Random Forest classifier model containing 40 features (including 37 bacterial species)	Loomba et al., 2017
Hepatic steatosis and fibrosis	Based on the link between the abundance of specific GM and 3-(4-hydroxyphenyl) lactate that shares a gene effect with hepatic steatosis and fibrosis	Caussy et al., 2018
NAFLD	Based on molecular networks linking the GM and the host molecular phenomics (hepatic transcriptome and plasma and urine metabolomes) to hepatic steatosis.	Hoyles et al., 2018

## Possible Mechanism of Gm’s Effect on the Occurrence and Prognosis of Nafld

The pathogenesis of NAFLD is thought to involve complex interactions among genetic susceptibility, environmental factors, insulin resistance, and changes in the GM. The “multiple-hit” hypothesis is adequate to explain the diverse metabolic and molecular changes observed in the development of NAFLD (Buzzetti et al., 2016; Fang et al., 2018). With the progress of metagenomics and non-targeted metabolomics, the role of the GM in the pathogenesis of NAFLD has attracted the attention of the scientific community. The GM plays an important role in the maintenance of host immune and inflammatory homeostasis, and the balance of nutrient intake and metabolism, thus directly or indirectly affecting the onset and development of NAFLD (Figure 1; Chu et al., 2019; Jennison and Byrne, 2021).

### Damage to the Intestinal Barrier and Aggravation of Secondary Inflammation

Emerging evidence shows that an intestinal barrier disorder leads to the translocation of the GM and metabolites, which can reach the liver directly along the gut–liver axis. Patients with NAFLD have decreased expression of junctional adhesion molecule A and zonula occludens-1, and increased intestinal permeability, which might be important factors in disease progression (Kolodziejczyk et al., 2019). Compared with simple hepatic steatosis, the correlation of increased intestinal permeability was stronger in patients with NASH, suggesting that inflammatory persistence and exacerbation might be caused by destruction of the intestinal barrier (Luther et al., 2015). In a mouse model, compared with the control group, the zonula occludens-1-deficient mouse group (intestinal barrier disorder model) fed a high fat, fructose, and cholesterol diet, developed more severe NASH (Rahman et al., 2016). Similarly, destruction of the intestinal barrier is considered an early event in the pathogenesis of NAFLD. Mice fed an HFD suffered from diet-induced intestinal dysbacteriosis after only 1week, resulting in intestinal barrier damage and bacterial translocation to the liver (De Santis et al., 2015; Mouries et al., 2019). Thus, an impaired intestinal barrier secondary to dysbacteriosis might be a prerequisite for diet-driven NAFLD.

It is unclear whether NAFLD is the cause or the result of intestinal barrier disruption. However, it can be confirmed that no matter what the triggering event is, the transfer of pro-inflammatory products, such as LPS, secondary to intestinal barrier damage, to the systemic circulation, will aggravate NAFLD and lead to poor prognosis (Gabele et al., 2011; Ipsen et al., 2018; Mouries et al., 2019). As shown in Figure 1B, excessive LPS translocation to the liver can interact with toll-like receptor (TLR)-4 on Kupffer cells and stellate cells, thus activating the nuclear factor kappa B (NF-κB) signaling pathway, and ultimately promoting the release of pro-inflammatory cytokines, such as interleukin (IL)-1, IL-6, and tumor necrosis factor, aggravating hepatic steatosis, inflammation, and fibrosis (Henao-Mejia et al., 2012; Plociennikowska et al., 2015; Carpino et al., 2020). TLR signaling in the mucosa also leads to the production of NOD-like receptor family, pyrin domain containing 3 (NLRP3), which results in the production of hepatic pro-inflammatory and pro-fibrotic mediators (e.g., caspase-1, IL-1β, and IL-18; Mridha et al., 2017; Carpino et al., 2020).

### Regulation of SCFAs in Metabolic and Inflammatory Pathways

Human SCFAs (e.g., acetate, propionate, and butyrate) are generated mainly from the fermentation of polysaccharides by the GM, and play a pivotal role in energy metabolism and inflammation regulation (Gomes et al., 2018). The different phenotypes of the GM and different dietary factors will affect the type and quantity of SCFAs synthesized in the gut. A high-fiber or resistant starch diet, the *Mediterranean* diet, and the enrichment of specific bacteria, such as *Akkermansia municiphilla* (producing propionate), *Ruminococcus*, *Faecalibacterium*, and *Eubacterium* (producing butyrate), can induce SCFA production (Morrison and Preston, 2016; Gomes et al., 2018).

Many polysaccharides cannot be hydrolyzed by the host, but can be realized by specific microbiota, finally generating SCFAs. If the excess SCFAs are not metabolized by colon cells, they will enter the liver and peripheral circulation through the portal vein, where they can be used as the substrates for fat synthesis and glycogenesis (Rau et al., 2018). This enables the host to obtain excess energy from food more efficiently, and to synthesize and store more fat to the liver (Harris et al., 2020). In a cohort study, with the development of NAFLD, higher abundances of SCFA-producing bacteria and intestinal acetate and propionate levels were observed (Rau et al., 2018). Interestingly, elevated peripheral levels of pro-inflammatory T cells (lower numbers of resting regulatory T-cells and higher numbers of Th17 cells) were observed simultaneously, which suggested that SCFAs are involved in the development of NAFLD, not only by affecting metabolism, but also by influencing immune and inflammatory responses (Rau et al., 2018). NAFLD is most associated with obesity. In mouse models and human studies, obese subjects have more carbohydrate metabolism genes in the intestinal microbiome and a higher concentration of SCFAs in the cecum, indicating that their production is excessive or their absorption is disrupted (Schwiertz et al., 2010). Zhao et al. (2020) indicated that liver lipid synthesis triggered by dietary fructose is dependent on metabolizing fructose to acetic acid and then to Acetyl Coenzyme A through the GM rather than *via* ATP citrate lyase. The hepatic metabolism of fructose promotes the transcription of genes related to hepatic lipid synthesis, and the metabolite acetate provides the raw material for this process. In addition, SCFAs can induce differentiation of T-cells into Th1 or Th17 cells, depending on the cytokine milieu and the epigenetic activity of histone deacetylases. IL-17 secreted by Th17 cells might play a pro-HCC role by promoting tumor angiogenesis (Liao et al., 2013; Park et al., 2015).

However, emerging evidence suggests the potential protective effect of SCFAs in NAFLD. Sodium butyrate can alleviate HFD-induced intestinal dysbacteriosis and endotoxemia, and thus inhibit NAFLD, by regulating intestinal and liver immune responses (Zhou et al., 2017a). One of the mechanisms is to affect the nutrient intake and metabolism of the host. Butyrate and propionate can activate free fatty acid receptor-3, and thus upregulate the production of the hormones intestine peptide YY and glucagon-like peptide (GLP)-1, which can increase satiety and reduce the intake of energy (Lin et al., 2012). Similarly, activation of GLP-1 has been proven to contribute to the recovery of hepatocyte function, the inhibition of hepatic steatosis and fibrosis, and the prevention of NAFLD developing into NASH (Tang et al., 2015a). SCFAs can also inhibit insulin signal transduction in adipocytes by activating G-protein receptor-43, thereby promoting glucose and unbound lipid metabolism, and inhibiting fat accumulation in liver and adipose tissue (Kimura et al., 2013). Another possible mechanism of SCFAs limiting NAFLD is to maintain a healthy intestinal barrier and to weaken inflammatory signals. SCFAs can prevent intestinal mucosal atrophy mediated by GLP-2 (Cani et al., 2009). Increasing the level of GLP-2 through microbial intervention can reduce the intestinal permeability and the levels of LPS and cytokines, thus reducing oxidative stress and liver inflammation (Cani et al., 2009). SCFA supplementation also showed beneficial effects in several inflammatory diseases (e.g., colitis; Rau et al., 2018). By inhibiting colitis, the intestinal barrier can be improved, thereby reducing the liver damage caused by bacterial translocation and the liver transfer of metabolites.

The effects of SCFAs are diverse and extensive, and different kinds and contents of SCFAs in different hosts show different and even contradictory biological effects; therefore, it is difficult to clarify their overall impact (Harris et al., 2020; Martin-Gallausiaux et al., 2020). In view of the close and complex relationship between SCFAs and host nutrient intake and metabolism, inflammation, and immunity, an in-depth study is needed to determine the specific mechanism by which SCFAs affect the occurrence and development of NAFLD.

### Regulation of Abnormal Cholesterol and BA Metabolism Mediated by Diet and the GM

Lipotoxicity promotes the progression of NASH, fibrosis, cirrhosis, and even HCC (Ioannou, 2016). Among liver lipids, cholesterol is the most important lipotoxic molecule in the development of NAFLD (Ioannou, 2016). Abnormal liver cholesterol homeostasis has been confirmed in both animal models and in humans with NASH. Zhang et al. (2021) revealed the GM-mediated mechanism of dietary cholesterol leading to the progression of NASH, that is, long-term high dietary cholesterol can induce an increase in taurocholic acid and the decrease of 3-indolepropionic acid by changing the GM (decreased levels of *Bifidobacterium* and *Bacteroides* and increase levels of *Desulfovibrionaceae Anaerotruncus*, *Desulfovibrio*, and *Mucispirillum*), thus promoting liver lipid accumulation and cell proliferation, leading to the occurrence of NAFLD-HCC (Zhang et al., 2021). In a mouse model, anti-cholesterol treatment eliminated completely the onset of NAFLD-HCC induced by dietary cholesterol (Zhang et al., 2021). This suggests that some of the mechanisms remain unknown: how the related pathogenesis inducing factor (e.g., HFD) causes inflammation, and how to accelerate the transformation of simple hepatic steatosis to NASH, which might be explained by the GM and its metabolites.

BAs are synthesized from cholesterol in the liver and play an important role in the digestion, absorption, and metabolism of fat. The GM is involved in the transformation and metabolism of BAs (Sanchez, 2018). The interaction between BAs and the GM plays an important role in the pathogenesis of NAFLD (Chiang and Ferrell, 2020). BAs participate in the pathogenesis of NAFLD through the farnesoid X receptor (FXR). By binding to FXR, BAs increase insulin sensitivity and reduce hepatic gluconeogenesis and triglyceride in the circulation (Chiang and Ferrell, 2020). Under the intervention of an HFD, the GM promotes weight gain and liver steatosis in an FXR-dependent manner, and the improvement of hepatic steatosis associated with antibiotic therapy depends on FXR signal transduction (Jiang et al., 2015; Parseus et al., 2017). In a large cohort of patients with NASH, although *Obecholate* (an FXR agonist) did not improve NASH, it significantly improved liver fibrosis compared with that in the control group (Neuschwander-Tetri et al., 2015). BAs also activate Takeda G-protein-coupled receptor 5 (TGR5) in muscle and adipose tissue, thereby increasing energy expenditure (Pols et al., 2011). In addition, activation of TGR5 in the intestine can promote the release of GLP-1, and then positively regulate the secretion of insulin (Pols et al., 2011; Kumar et al., 2016). TGR5 is also expressed in Kupffer cells, which are involved in the regulation of liver inflammation. Activation of TGR5 seems to induce anti-inflammatory effects by inhibiting the NF-κB signaling pathway and cytokine production (Perino and Schoonjans, 2015). The BA levels in liver, serum, and urine were increased in patients with NAFLD (Arab et al., 2017). In a phase II clinical trial in patients with NASH, the BA synthesis inhibitor, *Aldafermin*, reduced liver inflammation, steatosis, and fibrosis significantly (Harrison et al., 2021). The GM is likely to affect the BA pool, and regulates the metabolism of host cells through the transformation of BAs, including the homeostasis of lipids and glucose; however, its role in the pathogenesis of NAFLD remains controversial, which requires further in-depth study.

### Regulation of Choline and Its Derivatives

Choline deficiency is closely related to the induction and promotion of NAFLD, and is often used to construct animal models of NAFLD (Sherriff et al., 2016). Compared with those in the healthy group, patients with NAFLD generally showed lower levels of serum choline and higher levels of trimethylamine (TMA; Sherriff et al., 2016). In the absence of choline in human body (e.g., because of a choline deficient diet or gut dysbiosis), the synthesis of phosphatidylcholine is insufficient and the level of very-low-density lipoprotein is downregulated, which leads to liver lipid transfer disorder, enhanced mitochondrial β-oxidative damage, and oxidative stress in hepatocytes, eventually leading to liver steatosis, and aggravating liver inflammation and fibrosis (Smallwood et al., 2016). Some intestinal bacteria (e.g., *Desulfovibrio desulfuricans* and *Escherichia coli*) can convert choline to TMA and then to trimethylamine-N-oxide (TMAO) in the liver, which reduces the bioavailability of choline (Sohlenkamp et al., 2003). In addition, TMAO can promote insulin resistance by destroying blood glucose homeostasis and increasing the level of serum inflammatory cytokine C-C motif chemokine ligand 2 (CCL2), and affect lipid metabolism and BA homeostasis by reducing the conversion of cholesterol to BAs, which suggests that TMAO might affect NAFLD indirectly (Tang et al., 2015b). In fact, strategies to reduce TMA and/or TMAO have been used in the clinical treatment or prevention of NAFLD. For example, 3,3-dimethyl-1-butanol, a structural analog of choline, inhibits TMA and TMAO production by inhibiting microbial TMA lyase. However, based on the different microbial characteristics of individuals, it might only be effective for some patients (Wang et al., 2015). Therefore, additional genotyping of the NAFLD cohort is needed to identify patients that would respond to TMA and/or TMAO inhibitors.

### Regulation of Other Metabolites

Endogenous ethanol is produced by some intestinal bacteria *via* carbohydrate fermentation. Although obese mice with NAFLD did not ingest any alcohol, alcohol could still be detected in their breath (Cope et al., 2000). Compared with healthy individuals or patients with simple hepatic steatosis, the blood ethanol concentration in patients with NASH is higher, which is associated with increased liver inflammation and liver damage (Baker et al., 2010). Recent studies revealed that about 60% of patients with NAFLD have high alcohol producing *K. pneumoniae* in their intestines and their abundance is related to the severity of the disease (Yuan et al., 2019). The pathogenesis of NAFLD caused by endogenous ethanol is similar to that of alcoholic fatty liver disease (Parlesak et al., 2000; Seitz et al., 2018; Jennison and Byrne, 2021): (1) the induction of mitochondrial damage and enhanced oxidative stress; (2) destruction of the intestinal barrier and aggravation of liver damage through the gut–liver axis; (3) the induction of cytokines, chemokines, Th17, and other immune cells to intensify liver inflammation; and (4) the induction of liver cell damage through acetaldehyde-mediated cytotoxicity, metabolic disorder, and fat accumulation. These findings not only explain many of the similarities of the pathological features between the two diseases, but also provide a feasible method for clinical diagnosis and treatment of fatty liver caused by such bacteria (Brown and Kleiner, 2016).

Phenylacetic acid (PA) is mainly produced by the metabolism of aromatic amino acids (e.g., phenylalanine) by *Bacteroides* (Cook, 2019). A multi-omics study showed that PA levels were high in the serum of patients with NASH. At the same time, aromatic amino acids and branched chain amino acids increased, and bacterial diversity decreased (Hoyles et al., 2018). These characteristics have great potential as biomarkers for the clinical diagnosis and prediction of this disease.

Indole, one of the products of tryptophan metabolism by the GM, is generally considered to have anti-inflammatory effects (Yang et al., 2020). Clinical sample analysis, and mouse and cell experiments, showed that indole correlated negatively with NAFLD. Indole supplementation could reduce diet-induced NAFLD, liver fat accumulation, and the inflammatory response in mice. This protective effect was mediated by 6-phosphofructo-2-kinase/fructose-2,6-biphosphatase 3 (PFKFB3), a glycolysis regulatory factor of bone marrow cells (Ma et al., 2020). Mimicking or specifically activating PFKFB3 expression in macrophages using indole might be a feasible method to prevent and treat NAFLD and other inflammatory related diseases.

## Targeting the Gm As a Potential Strategy To Diagnose and Treat Nafld

At present, no effective or targeted drug for NAFLD has been approved for marketing. Lifestyle change is still the main intervention for NAFLD; however, the effect and patients’ compliance are poor. The close relationship between the GM and NAFLD has been confirmed. Intestinal dysbacteriosis and disturbance of metabolites (type, content, and proportion) and the subsequent metabolic, immune, and inflammatory homeostasis damage might be critical factors for NAFLD development. Thus, targeting the GM is a growing and promising field aiming to slow down and even reverse NAFLD (Sharpton et al., 2021). Moreover, based on the specific changes of the microbiota and metabolites in patients with NAFLD, the GM is also expected to be developed as non-invasive biomarker for the diagnosis, staging, and prognosis of NAFLD (Sharpton et al., 2021). Many studies have investigated the feasibility of treating NAFLD by altering the contribution of GM to its pathogenesis, including regulation by fecal microbiota transplantation, probiotics, prebiotics, and synbiotics (Kolodziejczyk et al., 2019). Notably, anti-LPS immunoglobulin, drugs to reverse the BA imbalance in NAFLD (e.g., FXR agonists, peroxisome proliferator activated receptor gamma (PPARα) agonists, and ursodeoxycholic acid), and drugs to restore intestinal barrier function and inhibit liver inflammation (e.g., butyrate) have shown encouraging therapeutic effects (Adar et al., 2012; Sun et al., 2018; Yu et al., 2018). Despite the exciting results in many animal studies, the results of multicenter human clinical trials with large samples are still needed. Some representative studies are summarized in Tables 2 and 3.

**Table 3:** Research on the treatment of NAFLD by targeting GM.

Intervention factors		Methods	Treatment results	References
Probiotics (traditional)	*B. longum*	Clinical trials	Hepatic steatosis↓	Malaguarnera et al., 2012
	*Probiotics VSL#3*	Clinical trials	BMI↓, hepatic steatosis↓, hepatic fibrosis↓	Alisi et al., 2014
	*Lepicol probiotic*	Clinical trials	Hepatic triglyceride↓, AST↓	Wong et al., 2013b
	*Parabacteroides distasonis*	Pre-clinical trials	Weight↓, bile acid, lipid and glucose metabolism homeostasis↑	Wang et al., 2019
	*B. xylanisolvens*	Pre-clinical trials	Hepatic butyrate and folate↑, Fat in liver and blood↓	Qiao et al., 2020
	*L. plantarum* NCU116	Pre-clinical trials	ALT↓, AST↓, lipogenesis↓, fatty acid oxidation↑	Li et al., 2014
	*L. acidophilus*	Clinical trials	ALT↓, AST↓	Abdel Monem, 2017
	*L. acidophilus* La5, *B. lactis* Bb12	Clinical trials	ALT↓, AST↓, LDL-C↓	Nabavi et al., 2014
	*L. acidophilus*, *L. rhamnosus*, *B. lactis*, *B. bifidum*	Clinical trials	Hepatic steatosis↓, TG↓, cholesterol↓,	Famouri et al., 2017
	*L. acidophilus*, *L. rhamnosus*, *L. paracasei*, *P. pentosaceus*, *B. lactis*, *B. breve*	Clinical trials	Total body fat↓, TG↓, intrahepatic fat↓	Ahn et al., 2019
	*L. bulgaricus*, *S. thermophilus*	Clinical trials	ALT↓, AST↓	Aller et al., 2011
	*L. casei*, *L. acidophilus*, *L. rhamnosus*, *L. bulgaricus*, *B. breve*, *B. longum*, *S. thermophilus*	Clinical trials	Insulin resistance↓, TNF-α↓, IL-6↓	Sepideh et al., 2016
	*L. johnsonii* BS15	Pre-clinical trials	Hepatic steatosis↓, ALT↓, TG↓, TNF-α↓	Xin et al., 2014
	*L. paracasei*	Pre-clinical trials	Hepatic steatosis↓, ALT↓, TLR4↓, TNF-α↓	Sohn et al., 2015
	*L. paracasei* N1115	Pre-clinical trials	Hepatic steatosis↓, TNF-α↓	Yao et al., 2019
	*L. reuteri* GMNL-263	Pre-clinical trials	Hepatic steatosis↓, Liver fibrosis↓, TGF-β↓	Ting et al., 2015
	*L. rhamnosus* GG	Pre-clinical trials	Hepatic fat content↓, TG↓, cholesterol↓	Kim et al., 2016
		Clinical trials	TNF-α↓, LPS↓	Bajaj et al., 2014
	Protexin	Clinical trials	ALT↓, AST↓, cholesterol↓, TG↓, BMI↓	Shavakhi et al., 2013
	Saccharomyces boulardii	Pre-clinical trials	AST↓, endotoxin↓, TNF-α↓, occludin↑	Liu et al., 2016
*Probiotics (novel)*	*A. muciniphila*	Pre-clinical trials	Hepatic inflammation↓, propionate↑, acetate↑, TG↓, insulin resistance↓	Cani and de Vos, 2017; Moreira et al., 2018
	*Bacteroides* spp.	Pre-clinical trials	BMI↓, propionate↑, acetate↑, TG↓	Tan et al., 2019
	*F. prausnitzii*	Pre-clinical trials	Intestinal integrity↑, hepatic steatosis↓, hepatic inflammation↓	Munukka et al., 2017
		Clinical trials	Butyrate↑, insulin resistance↓	Hippe et al., 2016; Bjorkqvist et al., 2019
	*Roseburia*	Clinical trials	Butyrate↑, pro-inflammatory cytokines↓	Louis and Flint, 2009
		Pre-clinical trials	Weight↓	Neyrinck et al., 2012
*Prebiotics*	Fructooligosaccharide	Clinical trials	Hepatic steatosis↓	Bomhof et al., 2019
	Inulin	Clinical trials	SCFA↑, pro-inflammatory cytokines↓	Bindels et al., 2012; Chambers et al., 2019
	Indole	Pre-clinical trials	Hepatic steatosis↓	Ma et al., 2020
*Synbiotics*	Synbiotic 2000Forte	Pre-clinical trials	LPS↓hepatic fibrosis↓	Cortez-Pinto et al., 2016
	*L. reuteri* and guar gum and inulin	Clinical trials	Hepatic steatosis↓, BMI↓	Ferolla et al., 2016
	*B. longum* and fructooligosaccharide	Clinical trials	Hepatic steatosis↓, LPS↓ insulin resistance↓, pro-inflammatory cytokines↓	Malaguarnera et al., 2012
*Fecal microbiota transplantation*	Standard diet mice to NASH mice	Pre-clinical trials	Hepatic steatosis↓, LPS↓, butyrate↑, Intestinal integrity↑	Zhou et al., 2017b
	Healthy and lean donors to NAFLD acceptor	Clinical trials	α-Diversity↑, butyrate↑insulin resistance↓	Vrieze et al., 2012

*L., Lactobacillus; S., Streptococcus; B., Bifidobacterium; P., Pediococcus; ALT, alanine amino transferase; AST, aspartate aminotransferase; LDL-C, low-density lipoprotein cholesterol; TG, triglyceride; TNF, tumor necrosis factor; and BMI, body mass index*.

## Conclusion and Prospects

Along with the lifestyle changes (excessive energy intake and reduced physical activity), NAFLD and its related diseases have become a global epidemic (Younossi, 2019; Huang et al., 2021). It is estimated that the morbidity of NASH will increase by as much as 56% in the next 10 years, and the incidence of NAFLD-HCC will double by 2030 (Huang et al., 2021). The initiation and progression of NAFLD have been proven to be the liver manifestation of disordered metabolic and immune homeostasis, which may be affected directly or indirectly by GM (Buzzetti et al., 2016; Fang et al., 2018). In recent years, research on the pathogenesis of NAFLD has made breakthroughs; and the advances in GM research have been deepening our understanding of NAFLD, and driving novel diagnostic and therapeutic approaches. However, the complex mechanism of the interaction between the GM and NAFLD has been illusive and limiting clinical progress (Neuschwander-Tetri, 2017; Fang et al., 2018). Whether alterations of the GM and its metabolites are driving factors or a consequence of the development of NAFLD should be further determined in the future.

With the rapid development of next-generation sequencing technology, metagenomics, and non-targeted metabolomics, we have made considerable progress in analyzing the composition and key metabolites of the GM, which has been considered as a potential and valuable non-invasive biomarker to diagnose NAFLD (Ebrahimzadeh Leylabadlo et al., 2020). However, different studies show different and even opposite results (Table 1). In addition, the use of the GM as a biomarker has inherent limitations: it is a highly dynamic aggregate, which is affected, for example, by host genes, living environment, lifestyle, and drugs. Based on massive samples, clinical data, and the results of multi-omics analysis, the combination of dynamic big data and artificial intelligence analysis might produce more reliable information.

The complexity of NAFLD means that there is still no feasible method to reverse the disease process or prevent its occurrence. The clinical significance of specific GMs and metabolite changes associated with NAFLD remains unclear. The strategy of targeting the GM to reverse the adverse changes of NAFLD has several limitations. Each patient might be associated with different diseases, including obesity and diabetes, and might harbor different predisposing factors, such as genes, diet, and metabolic phenotypes (Wang and Malhi, 2018; Younossi, 2019). A key breakthrough in the future will be the systematic integration of the manifestations, gene expression differences, GM, and metabolic differences in patients with different subtypes of NAFLD. Based on different phenotypes and the application of new technologies to precisely intervene with specific microbiota, it will provide new insights and more accurate treatment for NAFLD. Therefore, using probiotics and prebiotics to fight NAFLD blindly is not recommended until the role of the GM in the pathogenesis of NAFLD is further revealed. It is necessary to understand the functional interactions between the whole microbial community and NAFLD, thus further well-designed clinical trials and evidence-based medical data are needed.

## Author Contributions

All authors listed have made a substantial, direct and intellectual contribution to the work, and approved it for publication.

## Funding

This work was supported by the National Natural Science Foundation of China (31960236 and 31770536). Lanzhou Chengguan District Science and technology planning project (2020SHFZ0029); Lanzhou talent innovation and Entrepreneurship Project (2019-RC-34); Fund of the first hospital of Lanzhou University (ldyyyn2019-75).

## Conflict of Interest

The authors declare that the research was conducted in the absence of any commercial or financial relationships that could be construed as a potential conflict of interest.

## Publisher’s Note

All claims expressed in this article are solely those of the authors and do not necessarily represent those of their affiliated organizations, or those of the publisher, the editors and the reviewers. Any product that may be evaluated in this article, or claim that may be made by its manufacturer, is not guaranteed or endorsed by the publisher.

## Abbreviations

GM: Gastrointestinal microbiome
NAFLD: Non-alcoholic fatty liver disease
NASH: Non-alcoholic steatohepatitis
HCC: Hepatocellular carcinoma
BAs: Bile acids
SCFAs: Short chain fatty acids
LPS: Lipopolysaccharide
HFD: High fat diet
TLRs: Toll-like receptors
NLRP3: NOD-like receptor family, pyrin domain containing 3
GLP: Glucagon-like peptide
TGR5: Takeda G-protein-coupled receptor 5
TMA: Trimethylamine
TMAO: Trimethylamine-N-oxide
